# Structural and functional definition of the specificity of a novel caspase-3 inhibitor, Ac-DNLD-CHO

**DOI:** 10.1186/1471-2210-7-8

**Published:** 2007-06-27

**Authors:** Atsushi Yoshimori, Junichi Sakai, Satoshi Sunaga, Takanobu Kobayashi, Satoshi Takahashi, Naoyuki Okita, Ryoko Takasawa, Sei-ichi Tanuma

**Affiliations:** 1 Institute for Theoretical Medicine, Inc., Venture Kanda 504, 1-1-5 Uchikanda Chiyoda, Tokyo 101-0047, Japan; 2 Department of Biochemistry, Faculty of Pharmaceutical Sciences, Tokyo University of Science, 2641 Yamazaki Noda, Chiba 278-8510, Japan; 3 Genome and Drug Research Center, Tokyo University of Science, 2641 Yamazaki Noda, Chiba 278-8510, Japan; 4 Department of Molecular Biology, Faculty of Pharmaceutical Science, Tokushima Bunri University, 1314-1 Shido Sanuki, Kagawa 769-2193, Japan

## Abstract

**Background:**

The rational design of peptide-based specific inhibitors of the caspase family members using their X-ray crystallographies is an important strategy for chemical knockdown to define the critical role of each enzyme in apoptosis and inflammation. Recently, we designed a novel potent peptide inhibitor, Ac-DNLD-CHO, for caspase-3 using a new computational screening system named the Amino acid Positional Fitness (APF) method (*BMC Pharmacol*. 2004, 4:7). Here, we report the specificity of the DNLD sequence against caspase-3 over other major caspase family members that participate in apoptosis by computational docking and site-directed mutagenesis studies.

**Results:**

Ac-DNLD-CHO inhibits caspases-3, -7, -8, and -9 activities with K_i_^app ^values of 0.68, 55.7, >200, and >200 nM, respectively. In contrast, a well-known caspase-3 inhibitor, Ac-DEVD-CHO, inhibits all these caspases with similar K_i_^app ^values. The selective recognition of a DNLD sequence by caspase-3 was confirmed by substrate preference studies using fluorometric methylcoumarin-amide (MCA)-fused peptide substrates. The bases for its selectivity and potency were assessed on a notable interaction between the substrate Asn (N) and the caspase-3 residue Ser209 in the S_3 _subsite and the tight interaction between the substrate Leu (L) and the caspase-3 hydrophobic S_2 _subsite, respectively, in computational docking studies. Expectedly, the substitution of Ser209 with alanine resulted in loss of the cleavage activity on Ac-DNLD-MCA and had virtually no effect on cleaving Ac-DEVD-MCA. These findings suggest that N and L residues in Ac-DNLD-CHO are the determinants for the selective and potent inhibitory activity against caspase-3.

**Conclusion:**

On the basis of our results, we conclude that Ac-DNLD-CHO is a reliable, potent and selective inhibitor of caspase-3. The specific inhibitory effect on caspase-3 suggests that this inhibitor could become an important tool for investigations of the biological function of caspase-3. Furthermore, Ac-DNLD-CHO may be an attractive lead compound to generate novel effective non-peptidic pharmaceuticals for caspase-mediated apoptosis diseases, such as neurodegenerative disorders and viral infection diseases.

## Background

Apoptosis is a major form of cell death, characterized by a series of apoptosis-specific morphological alterations and nucleosomal DNA fragmentation of genomic DNA [1-3]. Recent studies toward understanding of the apoptosis machinery have revealed the essential roles of a family of cysteine aspartyl proteases named caspases (for reviews, refs 4 and 5). To date, 14 caspases have been implicated in the apoptotic and inflammatic pathway cascades: Caspases-2, -3, -6, -7, -8, -9, and -10 are involved in the initiation and execution of apoptosis, whereas caspases-1, -4, and -5 participate in the activation of pro-inflammatory cytokines during inflammation [4-9]. Apoptotic caspases can be subdivided into initiator and executioner caspases. They are normally expressed as proenzymes that mature to their fully functional form through proteolytic cleavage [4-9]. Autoprocessing of initiator caspases (e.g. caspases-2, -8, -9, and -10) is facilitated by adaptor proteins, such as the Fas-associated death domain protein (FADD) and apoptosis protease activating factor-1 (Apaf-1). Executioner caspases (e.g. caspases-3, -6, and -7) can be activated following proteolytic processing by initiator caspases [10,11]. Activated executioner caspases cleave a critical set of cellular proteins selectively and in a coordinated manner leading to cell death. More than 60 caspase substrates have been identified to date [12].

The caspase cascades in apoptosis maintain and amplify the original apoptotic stimuli, and their disregulations are involved as key factors in the development of a variety of diseases, including Alzheimers's disease [13], Parkinson's disease [14] and cancer [15]. In particular, caspase-3 has been characterized as the major contributor to the process of apoptosis, and the phenotype of caspase-3 knockout mice suggests the necessity of the enzyme during brain development [16]. Therefore, studies with peptide inhibitors of caspase-3 have helped to define a central role for the enzyme in apoptosis. So far, several peptide inhibitors of caspase-3 have been reported [17-20], some of which were effective in animal models of amyotrophic lateral sclerosis (ALS) [21], sepsis [22], and hypoxic-ischemic brain injury [23].

Among caspases, the structures of caspases-1, -2, -3, -7, -8, and -9 have been determined by X-ray crystallography [24-29]. The three-dimensional structures reveal that the active sites of all caspases contain positively charged S_1 _subsites that bind the negatively charged Asp in the P_1 _position on the substrates. Since the S_1 _subsites are highly conserved, all caspases cleave solely after aspartate residues [7,24-29]. Recognition of at least four amino acids (P_1_–P_4_) in the cleavage sites is also a necessary requirement for efficient catalysis. The S_2_–S_4 _subsites on caspases vary significantly, resulting in varied substrate specificities for the P_2_–P_4 _positions, despite an absolute requirement for Asp in the P_1 _position [7,24-29]. To define the peptide substrate specificities at the P_2_–P_4 _positions of caspases, a combinatorial approach using a positional scanning synthetic combinatorial library (PS-SCL) was taken. As a result, the optimal recognition sequence of peptide substrate for caspase-3 was shown to be DEVD [30]. The sequence DEVD within poly(ADP-ribose) polymerase (PARP) is known to be recognized and cleaved by caspase-3 [9]. This sequence has been applied to creating the peptide aldehyde inhibitor Ac-DEVD-CHO. However, Ac-DEVD-CHO inhibits not only caspase-3 activity, but also the activities of caspases-1, -6, -7, -8, -9, and -10 [31]. To date, therefore, no tetrapeptide inhibitor selective for caspase-3 has yet to be identified.

It should be possible to derive the most selective and potent peptide inhibitor for each caspase from its comprehensive tetrapeptide library. In this regard, we recently developed a new strategy named the APF method for the computational design of peptides with high affinities for target proteins [32]. This computational virtual screening method allows the rapid prediction of binding free energies between all peptides being tested and a target protein. Recently, we used this method to identify potent peptide inhibitors of caspase-3. Importantly, a novel specific peptide inhibitor, Ac-DNLD-CHO, was shown to have almost the same potent inhibitory activity against caspase-3 as the well-known Ac-DEVD-CHO. In this study, we investigated the structural and functional relevance to potency and selectivity of this rationally designed peptide by enzyme kinetic analyses, computational docking studies and site-directed mutagenesis analysis. The results identify the specific interaction of the P_3 _position (Asn) in DNLD with the S_3 _(Ser209) subsite on caspase-3 as the most important determinant of the selectivity of Ac-DNLD-CHO.

## Results

### Potency and selectivity of Ac-DNLD-CHO against caspase-3

**Figure 1:**
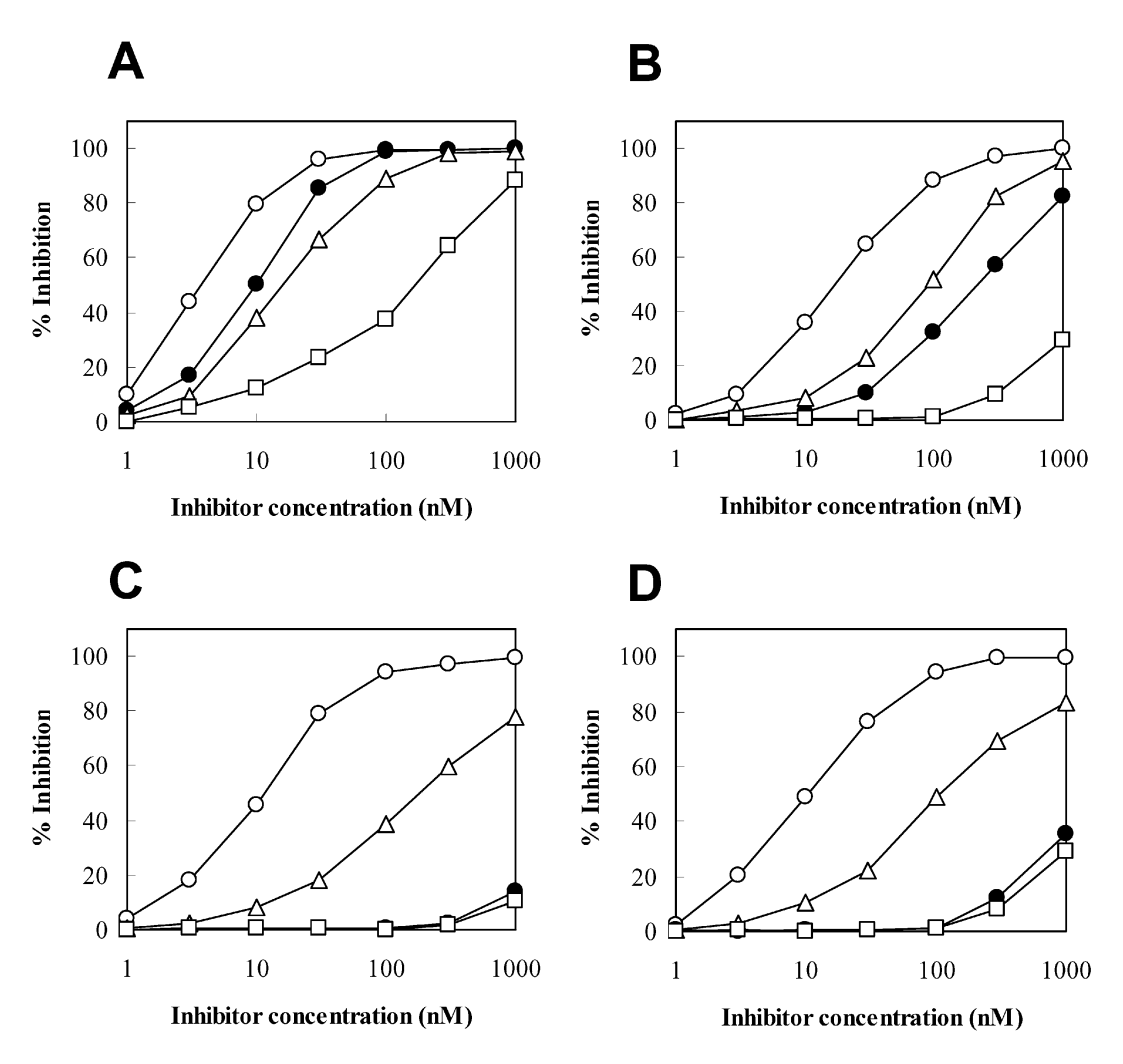
**Inhibitory effects of caspase-3 inhibitors on caspases**. Human recombinant caspase-3 (A), caspase-7 (B), caspase-8 (C), and caspase-9 (D) were preincubated for 10 min with indicated concentrations of Ac-DNLD-CHO (●), Ac-DEVD-CHO (○), Ac-DQTD-CHO (△), or Ac-DMQD-CHO (□), and then the activities of the caspases were measured with each substrate as described in ''Methods''. The kinetics data presented are the means of three independent experiments.

At present, three peptide inhibitors (aldehyde form) of caspase-3, Ac-DEVD-CHO, Ac-DQTD-CHO, and Ac-DMQD-CHO, are commercially available. Therefore, we compared the potency and selectivity of these peptide inhibitors with Ac-DNLD-CHO using human recombinant caspases-3, -7, -8, and -9. As shown Fig. 1A, Ac-DNLD-CHO inhibits caspase-3 (IC_50 _= 9.89 nM) with almost the same potency as Ac-DEVD-CHO (IC_50 _= 4.19 nM). The apparent K_i_^app ^values for Ac-DNLD-CHO and Ac-DEVD-CHO are calculated to be 0.680 and 0.288 nM, respectively (Table 1). Ac-DEVD-CHO inhibits caspase-7 (K_i_^app ^= 4.48 nM), -8 (K_i_^app ^= 0.597 nM), and -9 (K_i_^app ^= 1.35 nM) to similar extents. This observation is consistent with the previous report that Ac-DEVD-CHO inhibits caspases-3, -7, and -8 [31]. In contrast, Ac-DNLD-CHO has very low inhibitory activity against caspases-8 and -9. Although Ac-DNLD-CHO inhibits caspase-7, the IC_50 _(245 nM) is one-order of magnitude higher than that of Ac-DEVD-CHO (IC_50 _= 19.7 nM). It is noteworthy that Ac-DNLD-CHO exhibits an approximate by 80-fold selectivity for caspase-3 over caspase-7 (K_i_^app ^= 55.7 nM) (Table 1), although these caspases have very similar protein structures and substrate preferences [30].

**Table 1:** Potency and selectivity of caspase-3 inhibitors.

	K_i_^app ^^a ^(nM)			
	
Inhibitor	Caspase-3	Caspase-7	Caspase-8	Caspase-9
Ac-DEVD-CHO	0.288 ± 0.087	4.48 ± 0.21	0.597 ± 0.095	1.35 ± 0.31
Ac-DQTD-CHO	1.27 ± 0.11	21.8 ± 1.1	9.75 ± 1.09	14.5 ± 0.7
Ac-DMQD-CHO	13.3 ± 0.3	>200	>200	>200

Ac-DNLD-CHO	0.680 ± 0.163	55.7 ± 6.0	>200	>200

^a ^K_i_^app ^was calculated from the IC_50 _value, K_i_^app ^= IC_50_/(1+ [S]/K_m_), where K_m _is the Michaelis constant of the substrate and [S] is the substrate concentration in the assay.

Ac-DQTD-CHO inhibits caspases-3 (K_i_^app ^= 1.27 nM) and -8 (K_i_^app ^= 9.75 nM) weakly as compared to Ac-DEVD-CHO, and inhibits caspase-9 (K_i_^app ^= 14.5 nM) and -7 (K_i_^app ^= 21.8 nM) even more weakly (Fig. 1 and Table 1). These inhibition curves for Ac-DQTD-CHO are shifted about one order of magnitude lower than those of Ac-DEVD-CHO. On the other hand, Ac-DMQD-CHO inhibits caspase-3 weakly (IC_50 _= 193 nM), whereas it has little inhibitory effect on caspases-7, -8, and -9 even at concentrations up to about 200 nM (Fig. 1 and Table 1). The kinetic analyses show Ac-DNLD-CHO to have a potent and selective inhibitory activity against caspase-3, whereas Ac-DEVD-CHO potently inhibits all four caspases tested.

### Abilities of caspases to cleave the fluorometric substrate of DNLD

**Figure 2:**
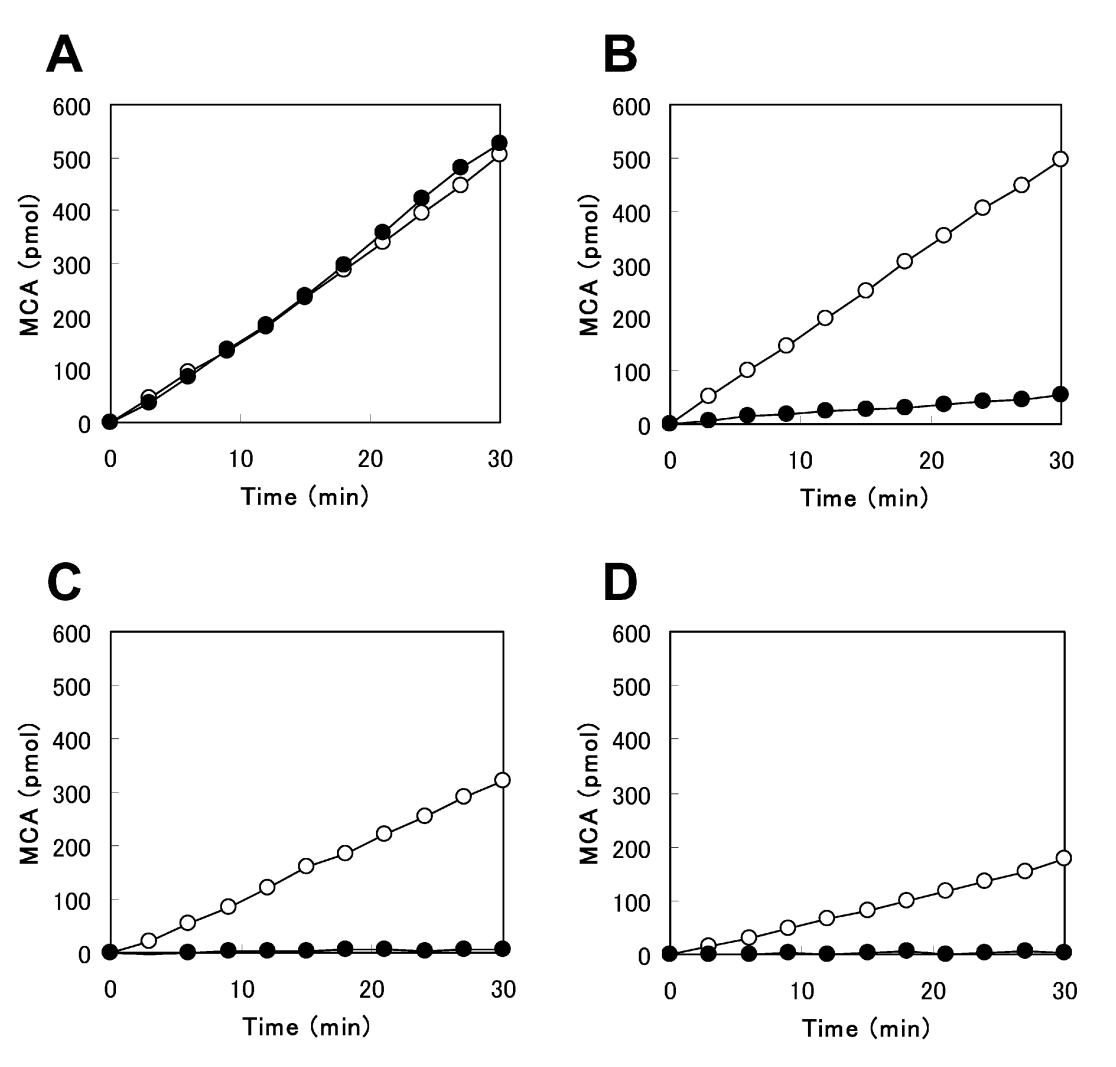
**Hydrolysis of Ac-DNLD-MCA by caspases**. The time course of the abilities of caspases-3 (A), -7 (B), -8, (C) and -9 (D) to cleave the fluorometric caspase substrates Ac-DNLD-MCA (●) and Ac-DEVD-CHO (○) were compared. The cleavage assay was performed as described in "Methods" The y-axis represents the concentration of MCA production (pmol) and the x-axis represents incubation period. Data indicate the mean of three independent experiments.

To determine the potency and selectivity of the DNLD sequence against caspase-3, we compared substrate preferences between DNLD and DEVD among caspases. Currently, Ac-DEVD-MCA is used as the substrate for caspase-3 (DEVDase). Unfortunately, caspases-7, -8, and -9 are also able to cleave this substrate (Fig. 2), consistent with the previous observation [33] that although caspase-3 is the major source of cellular DEVDase activity in apoptotic cell extracts, other caspases are considered to contribute to the DEVDase activity. That is, DEVDase is not necessarily equivalent to the true activity of caspase-3. From these data, it is clear that using Ac-DEVD-MCA as a substrate makes it hard to measure precisely the activity of caspase-3 alone in crude cell extracts.

To probe the functional difference between DNLD and DEVD sequences, we synthesized Ac-DNLD-MCA and examined its preference as a substrate. As shown in Fig. 2A, Ac-DNLD-MCA is cleaved as efficiently by caspase-3 as Ac-DEVD-MCA. Importantly, Ac-DNLD-MCA is hardly cleaved by caspase-7 (Fig. 2B). Additionally, caspases-8 and -9 have no ability to cleave Ac-DNLD-MCA (Fig. 2C and 2D). This implies that using Ac-DNLD-MCA makes it possible to measure the sole activity of caspase-3 in cell extracts. Taken together, these data suggest that the recognition of the DNLD sequence among the caspases is selective to caspase-3.

### Definition of active site residues of caspases

**Figure 3:**
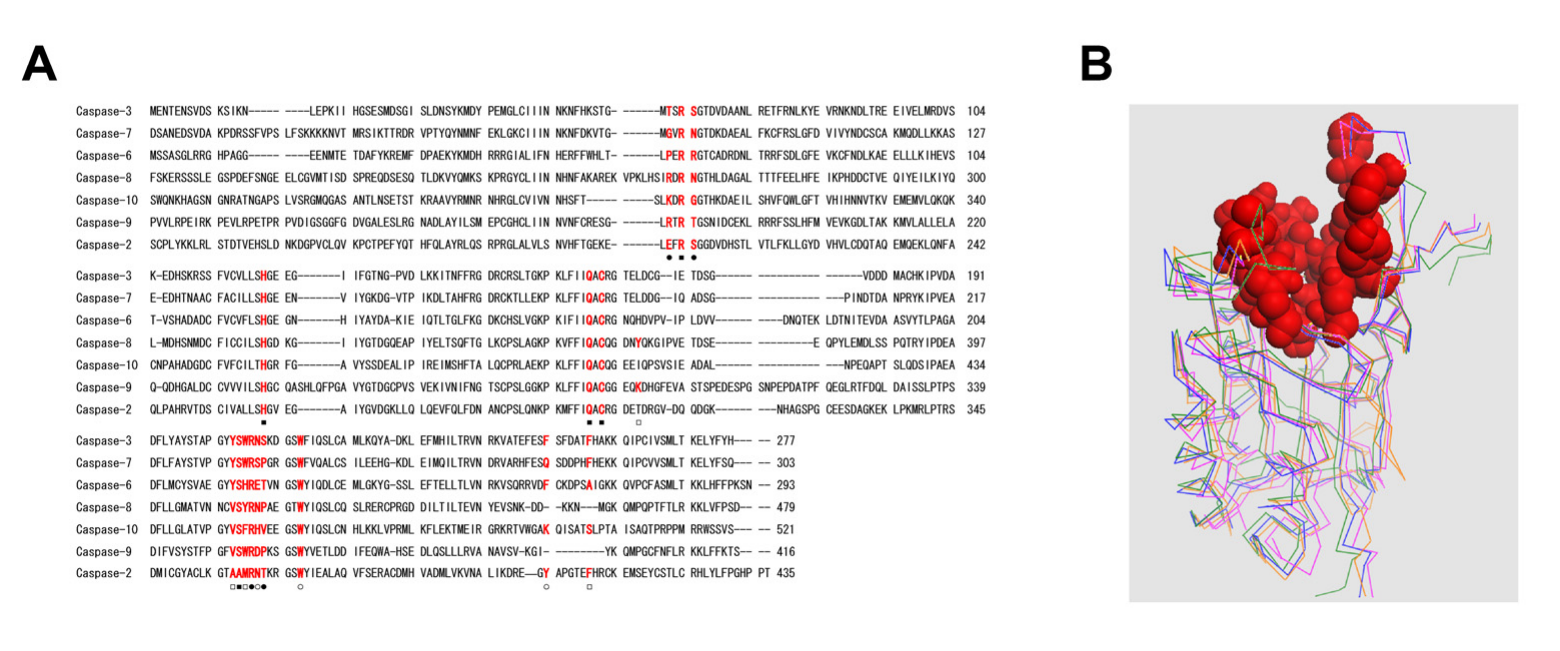
**Sequence alignments and structural superpositions of caspases**. A, Polypeptide sequence alignments of human caspases were performed using the Clustal W program [44] and then adjusted manually. Amino acid residues are numbered to the right of each sequence. Active site residues are highlighted in red, and S_1 _(■), S_2 _(□), S_3 _(●), and S_4 _(○) subsites on the active sites are indicated. B, Structural superpositions of Cα atoms of caspases-3 (blue), -7 (pink), -8 (green), and -9 (orange) are presented in line representation, and active site residues on the three-dimensional structures of caspase-3 are red in space-fill representation. The superpositions were performed using the McLaghlan algorithm [47] as implemented in the ProFit program [48].

**Figure 4:**
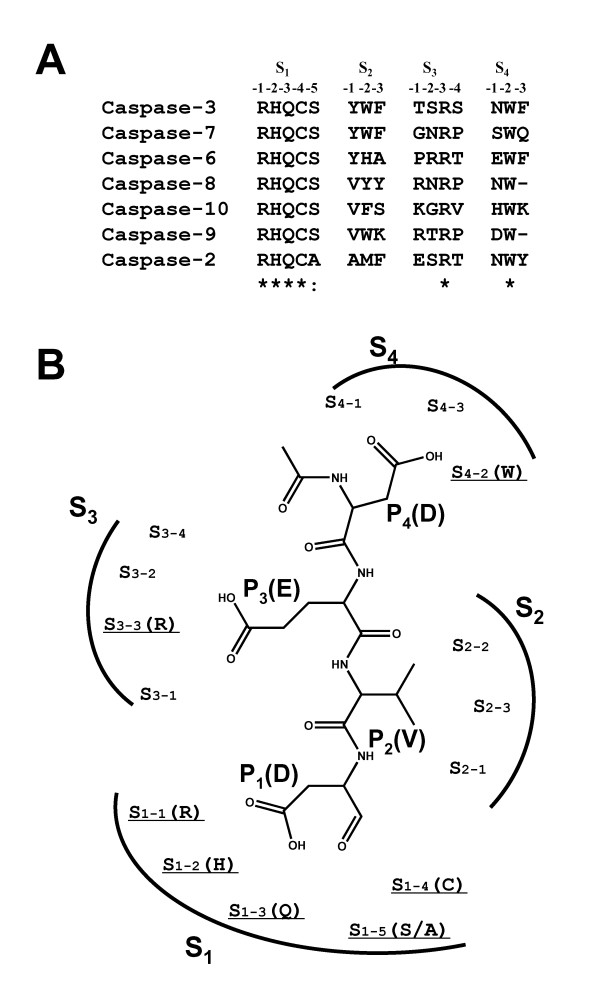
**Definition of active site residues of caspases**. A, Alignments of active site residues of caspases. The residues were identified by inspecting hydrogen bonding and van der Waals interactions in the X-ray structures of caspases (codes 1PAU, 1F1J, 1F9E, and 1JXQ) and using examples as described [27], and then assigning the particular subsites (S_x1-x2_, where x2 is the position in the S_x1 _subsite). B, Schematic representation showing the locations of the subsites on the active site with Ac-DEVD-CHO. The underlined subsite has a conserved residue except the S_1–5_(S/A) subsite.

In order to understand the specificity of the sequence DNLD for caspase-3 in the structural aspects, we next sought to determine the amino acid residues composing the active sites of caspases. By inspecting the interactions of peptide/caspase complexes in their X-ray structures [24-29], we identified fifteen and fourteen residues in the active sites of caspases-2, -3, -6, -7 and -10, and caspases-8 and -9, respectively (Fig. 3A). The active site residues are located in segments on the linear sequences of caspases (Fig. 3A), and construct similar 3 dimensional pockets of the active sites (Fig. 3B). From these data, 4 subsites (S_1_, S_2_, S_3_, and S_4_) (Fig. 4A) can be assigned that indicate common structural positions in the active sites of all caspases (Fig. 4B).

The aligned active site residues in each caspase construct almost the same subsites. The difference is that the active site residues (ϒ in Fig. 3A) of caspases-3 (F256), -7 (F282), -6 (A269), -10 (S500) and -2 (F409) belonging to the S_2–3 _subsites are aligned at the same position in the sequences, while those of caspases-8 (Y365) and -9 (K292) are aligned at different positions (compare Fig. 3A and Fig. 4). However, the structural superposition of these caspases (Fig. 3B) reveals that these amino acids are located at the same position (S_2–3_) in the S_2 _subsites (Fig. 4). Previous work has also shown that caspase-8 has Y365 situated in roughly the same spatial position as F256 of caspase-3 [27]. Thus, using the primary and tertiary structural information about caspases, we identified the amino acid residues important for substrate binding in the active sites of caspases as illustrated in Fig. 4 in a model of DEVD binding.

### Evaluation of active site definition

**Figure 5:**
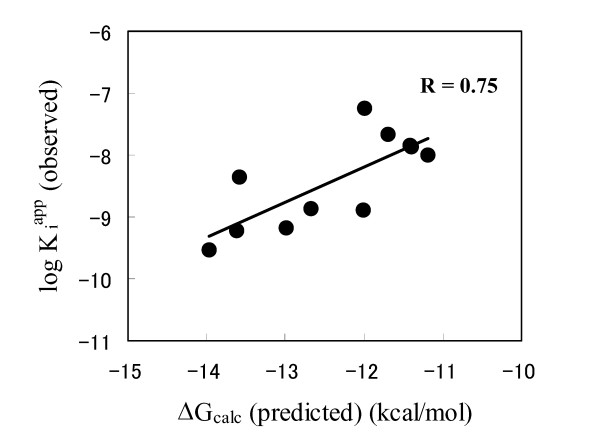
**Plot of the least squares fit plot of the predicted binding free energies (ΔG_calc_) (kcal/mol) vs experimentally observed K_i_^app ^values**. Least squares fit analysis performed over set of the data points in Table 2 yielded the following equation, log K_i_^app ^= +0.56ΔG_calc _- 1.43.

Fig. 5 shows the plots of the free energies of binding calculated by AutoDock [34] and the experimentally observed K_i_^app ^values summarized in Table 2. The best value obtained for Ac-DNLD-CHO is -12.99 kcal/mol (complex with caspase-3). Importantly, there are good correlations between all the calculated free energies of binding of Ac-DNLD-CHO to caspases-3, -7, -8 and -9, and their observed K_i_^app ^values. The correlation coefficient of R = 0.75 obtained from the plots is an appropriate value, suggesting that the AutoDock program used here produces reliable binding modes, and that the definition of amino acid residues composing the active sites of caspases is adequate.

**Table 2:** Comparisons of calculated free energies of binding and observed Ki values.

Caspase	Peptide Inhibitor (Ac-, -CHO)	ΔG_calc_(predicted) (kcal/mol) ^a^	K_i_^app ^(observed) (nM) ^b^	RMSD (Å) ^c^
3	DNLD	-12.99	0.68	-
	DEVD	-13.96	0.288	1.28
	DQTD	-12.01	1.27	-
	DMQD	-11.39	13.3	-

7	DNLD	-11.99	55.7	-
	DEVD	-13.59	4.48	1.41
	DQTD	-11.69	21.8	-
	DMQD	-11.85	>200	-

8	DNLD	-10.78	>200	-
	DEVD	-13.61	0.597	1.85
	DQTD	-11.19	9.75	-
	DMQD	-9.67	>200	-

9	DNLD	-9.98	>200	-
	DEVD	-12.68	1.35	1.46
	DQTD	-11.42	14.5	-
	DMQD	-9.58	>200	-

^a ^Binding free energies calculated by AutoDock [34].

^b ^Experimentally observed K_i_^app ^values from Table 1.

^c ^RMSD between docked and crystal or constructed structure.

Data points where K_i_^app ^> 200 nM were excluded from the least square fit plot in Fig. 5.

**Figure 6:**
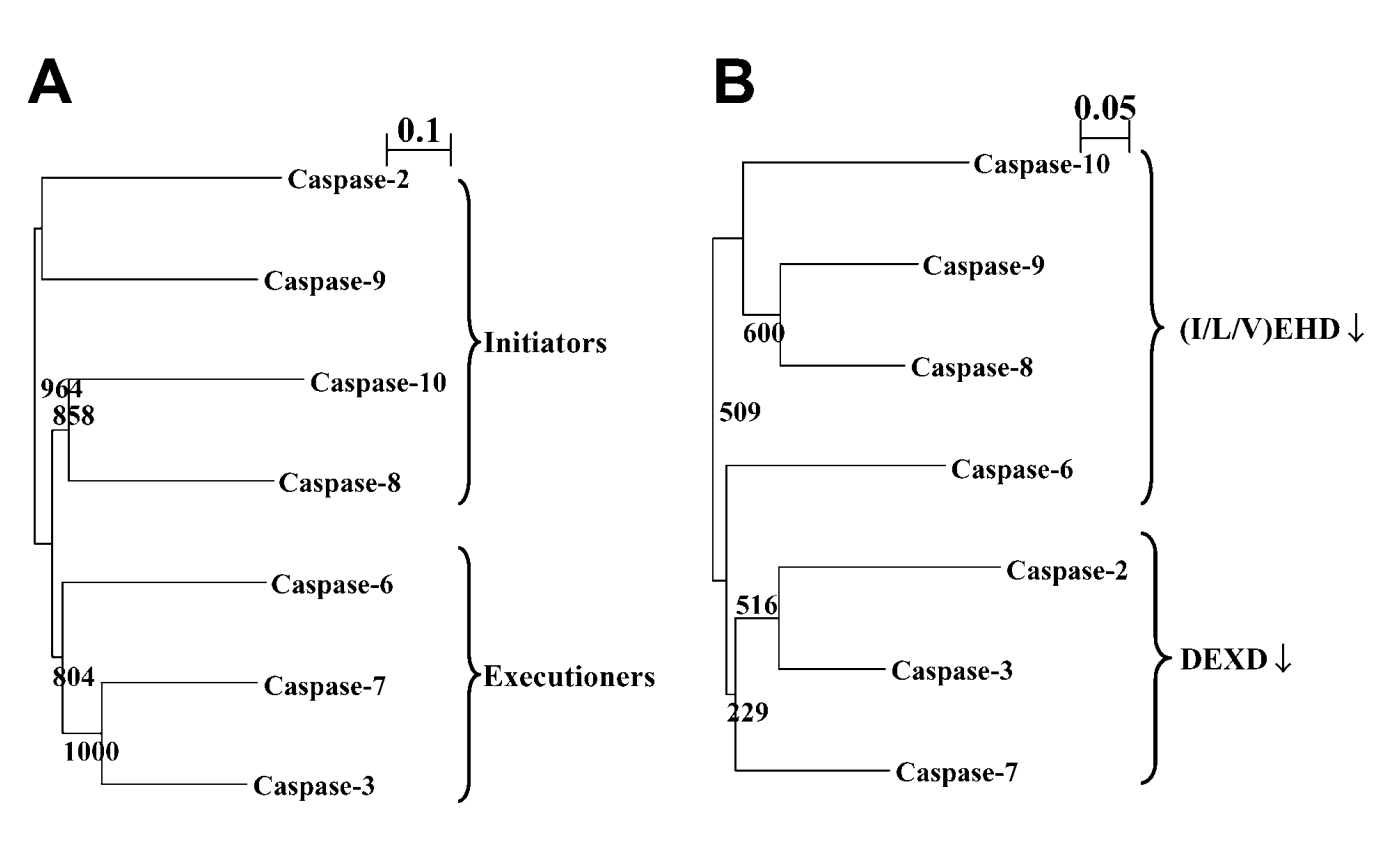
**Phylogenetic relationships of caspases**. Phylogenetic relationships were constructed by the neighbor-joining algorithm [35]. Bootstrap analysis was performed on 1000 random samples and analyzed by the Clustal W program [44]. The numbers at branches were determined by the bootstrap analysis, indicating the times in 1000 repeat samples. The relationships are based on full-length caspases (A) and the active site residues according to our definition (B).

Phylogenetic analysis of the apoptotic caspases-2, -3, -6, -7, -8, -9, and -10 shows that executioner caspases (-3, -7, and -6) belong to the same subfamily and initiator caspases (-2, -8, -9, and -10) belong to other subfamilies, thus reflecting their functional roles (Fig. 6A). If our active site definition is adequate, a similar phylogenetic analysis against the active site residues defined above (Fig. 4B) would reflect their substrate specificities. The phylogenetic analysis of the active site residues was conducted by the NJ algorithm [35]. Interestingly, the active site phylogeny is consistent with the substrate specificities of caspases (Fig. 6B). Nicholson and co-workers have clearly demonstrated that caspases are divided into three groups on the basis of substrate specificity analysis using a combinatorial approach: Group I enzymes (caspases-1, -4, and -5) prefer (W/Y)EHD↓ peptides; Group II enzymes (caspases-2, -3, and -7) prefer DEXD↓ peptides; Group III enzymes (caspases-6, -8, -9, and -10) prefer (I/L/V)EXD↓ peptides [30,31]. As shown in Fig. 6B, caspases-3, -7, and -2 (Group II) are classified into together, and other caspases-6, -8, -9, and -10 (Group III) fall into other classes. It should be noted that caspase-2 belongs to the same class as caspases-3 and -7 in our active site phylogenetic analysis (Fig. 6B), although caspase-2 is suggested to be an initiator caspase (Fig. 6A). The results strongly support the validity of our active site definition as illustrated in Fig. 4B.

### Evaluation of docking program for binding mode analysis

To elucidate the reason that Ac-DNLD-CHO is a potent and selective inhibitor for caspase-3 even though it has the same DXXD motif as Ac-DEVD-CHO, Ac-DQTD-CHO, and Ac-DMQD-CHO, computational docking studies were employed. We performed docking analysis using the AutoDock algorithm [34] to examine the binding modes of the peptide inhibitors at the active sites of caspases-3, -7, -8, and -9.

Among the above caspase inhibitors, a well-known Ac-DEVD-CHO was docked into the active sites of caspases, and then we measured how close the AutoDock can reproduce their complex structures of Ac-DEVD-CHO/caspase. Superpositions with the most minimized energies of docked Ac-DEVD-CHO in complexes of Ac-DEVD-CHO/caspases-3, -7, -8, and -9 yield root mean square distances (RMSDs) of 1.28 Å, 1.41 Å, 1.85 Å, and 1.46 Å, respectively (Table 2). Hence, AutoDock analysis is successful in reproducing the binding conformation of tetrapeptide inhibitors (aldehyde form). Furthermore, the results indicate that our docking procedures (energy minimization protocol and docking parameters) are reliable.

### Selective binding mode of Ac-DNLD-CHO with caspase-3

**Figure 7:**
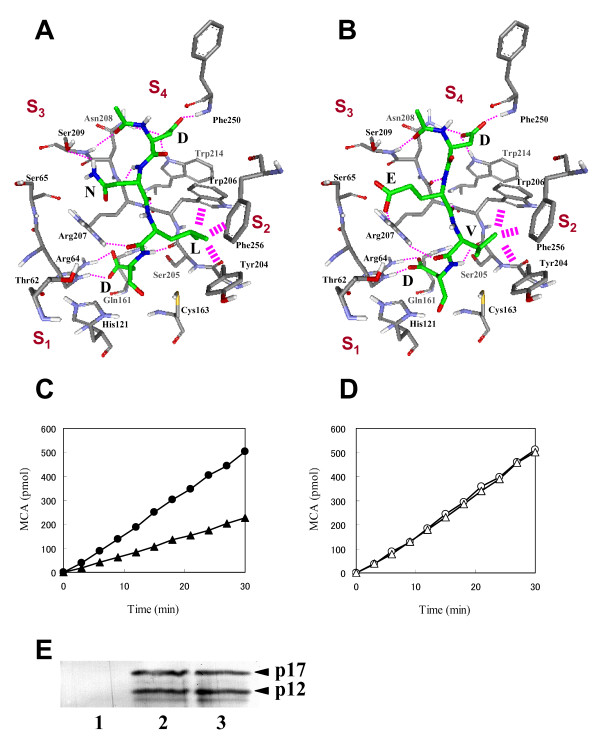
**Binding interactions for Ac-DNLD-CHO and Ac-DEVD-CHO on the active site of caspase-3**. Nitrogen, oxygen, and carbon atoms of the inhibitors are illustrated in blue, red, and green, respectively. Hydrogen bonds are shown as dashed lines. Hydrophobic interactions are shown as thick broken lines schematically. A, The binding mode of Ac-DNLD-CHO was obtained from docking simulations. B, The binding mode of Ac-DEVD-CHO was obtained from the X-ray crystal structure (1PAU). C, The time courses of liberation of fluorescence (MCA) from Ac-DNLD-MCA catalyzed by wild-type caspase-3 (●) and substituted (S209A) caspase-3 (▲). D, The time courses of liberation of fluorescence (MCA) from Ac-DEVD-MCA catalyzed by wild-type caspase-3 (○) and substituted (S209A) caspase-3 (△). The cleavage assays were performed as described in ''Methods''. Data indicate the mean of three independent experiments. E, Amounts of wild-type (lane 2) and substituted (S209A) (lane 3) active caspase-3 proteins generated by coexpression of HA-p17 and HA-p12 subunits in in vitro translation system were analyzed by Western blotting as described under ''Methods''. In this experiment, empty vector was used as control (lane 1).

To understand the binding mode of Ac-DNLD-CHO with caspase-3, Ac-DNLD-CHO was docked onto the active site of caspase-3 by the AutoDock program (Fig. 7A). The main-chain of Ac-DNLD-CHO forms hydrogen bonds at NH (Asn3) – O (Arg207), O (Asn3) – NH (Arg207), O (Leu2) – HH1 (Arg207), and NH (Asp1) – O (Ser205) in the S_1 _and S_3 _subsites, respectively (Table 3). Asp in the P_4 _position of Ac-DNLD-CHO donates hydrogen bonds to the Asp208, Trp214, and Phe250, and Asp in the P_1 _position interacts with Arg64. All of these interactions are observed in the complex of Ac-DEVD-CHO/caspase-3 although the hydrogen bonding distances and angles are slightly different (Table 3, Fig. 7B). It should be noted that Asn (P_3_) and Leu (P_2_) in Ac-DNLD-CHO have characteristic interaction patterns with caspase-3; the HD of Asn (P_3_) forms a direct hydrogen bond with OG of Ser209 (S_3–4 _subsite) and does not interact with Arg207 (S_3-3 _subsite), while Leu in the P_2 _position forms tight hydrophobic contacts with Trp206, Tyr204, and Phe256 in the S_2 _subsite of caspase-3. Meanwhile, the Glu in the P_3 _position of Ac-DEVD-CHO forms a direct interaction with Arg207 but not Ser209, although water-mediated interactions with Ser209 and/or Ser65 may exist (compare 7A and 7B, see Additional file 1)

**Table 3:** Comparisons of direct hydrogen bonding interactions between Ac-DNLD-CHO/caspase-3 and Ac-DEVD-CHO/caspase-3

Caspase-3			Ac-DNLD-CHO ^a^				Ac-DEVD-CHO ^b^			
Active site	Residues	Atom	Residues	Atom	Distance (Å)	Angle (deg)	Residues	Atom	Distance (Å)	Angle (deg)

S_4_	Asn208	HD2	Asp4	OD2	3.41	174.12	Asp4	OD1	3.23	157.09
	Trp214	HE1	Asp4	OD2	2.99	152.10	Asp4	OD1	3.78	150.54
	Phe250	HN	Asp4	OD1	3.22	136.14	Asp4	OD2	2.76	163.40

S_3_	Ser209	HN	Acetyl	O	3.10	144.37	Acetyl	O	2.84	167.94
		OG	Asn3	HD	2.69	116.54	-	-	-	-
	Arg207	HH2	-	-	-	-	Glu3	OE2	2.87	132.63
		O	Asn3	HN	2.82	141.89	Glu3	HN	2.77	155.73
		HN	Asn3	O	2.80	166.06	Glu3	O	2.78	165.67
		HH1	Leu2	O	2.93	141.53	Val2	O	3.26	141.52

S_1_	Arg64	HH2	Asp1	OD2	3.03	156.84	Asp1	OD1	2.72	169.65
		HE	Asp1	OD1	2.65	128.01	Asp1	OD2	2.56	164.98
	Ser205	O	Asp1	NH	2.66	170.78	Asp1	NH	2.84	142.41

Active site	Residues		Residues		ΔASA (Å^2^)^c^		Residues		ΔASA (Å^2^)^c^	

S_2_	Tyr204		Leu2		145		Val2		113	
	Trp206									
	Phe256									

^a ^Hydrogen bonding interactions of Ac-DNLD-CHO and caspase-3 were obtained from AutoDock docking simulations [34].

^b^Hydrogen bonding interactions of Ac-DEVD-CHO and caspase-3 were obtained from the crystal structure.

^c^The change in Accessible Surface Area (ASA) per Leu2 or Val2 residue upon dissociation of the complex structure was calculated by DSSP [54], and then mainly the results from the interactions with the S_2 _subsite. In calculating the ΔASA, it was assumed that no conformational changes in the peptide inhibitors or caspase-3 occur upon dissociation.

**Figure 8:**
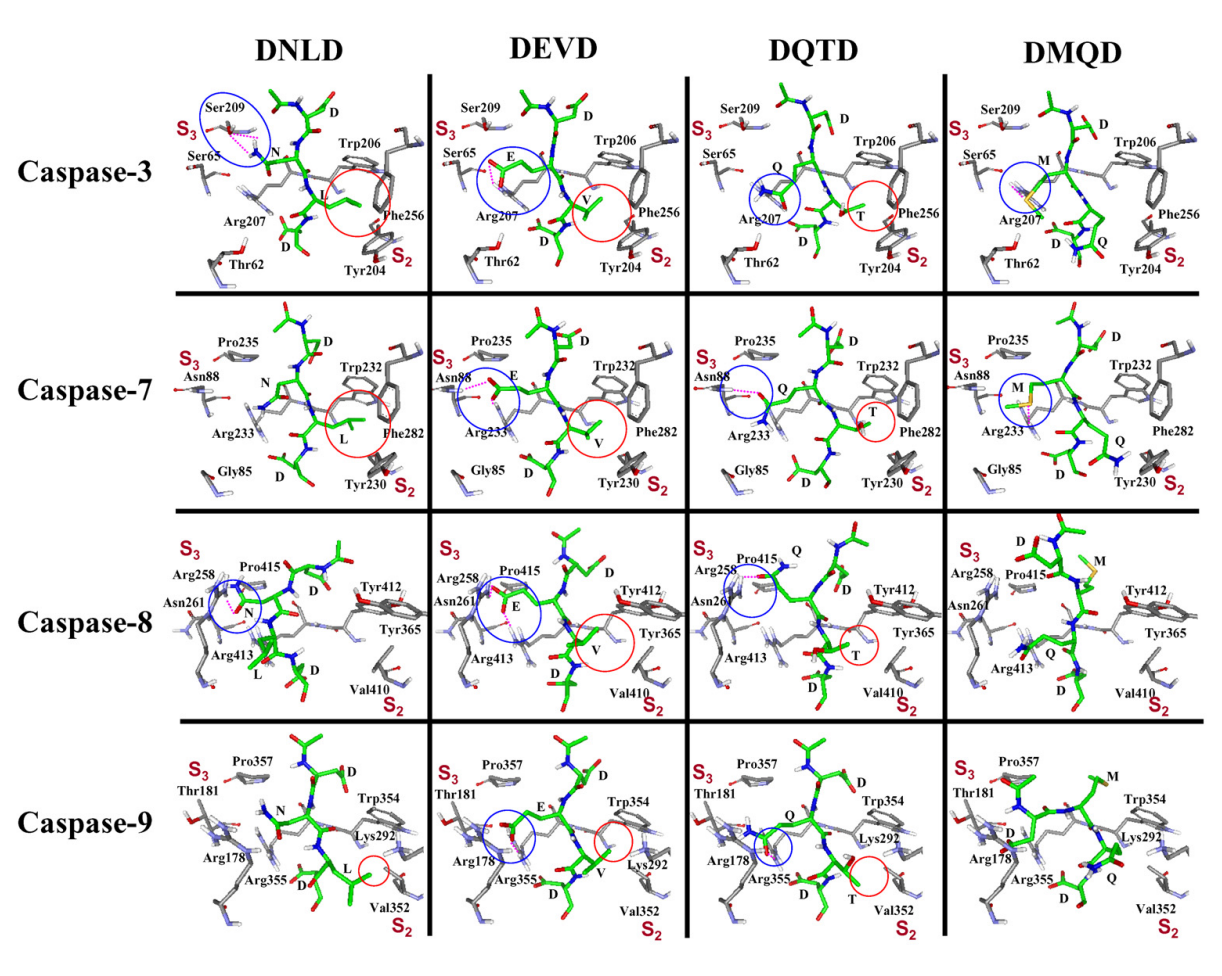
**Comparison of the binding modes of Ac-DNLD-CHO, Ac-DEVD-CHO, Ac-DQTD-CHO, and Ac-DMQD-CHO with caspases-3, -7, -8, and -9**. Nitrogen, oxygen, and carbon atoms of the inhibitors are illustrated in blue, red, and green, respectively. Hydrogen bonds with the S3 subsite are shown as blue circles and dashed lines. Hydrophobic interactions with the S2 subsite are shown as red circles.

Importantly, the S_3–4 _subsites of caspases-7, -8, and -9 have a conserved Pro residue (Fig. 4A). Consequently, a hydrogen bond between Asn (P_3_) in Ac-DNLD-CHO and the S_3–4 _subsite (Pro) of caspases-7, -8, and -9 can not be formed (Fig. 8, 1st. column). Furthermore, in the hydrophobic S_2 _subsites of caspases-8 (Val410, Tyr412, and Tyr365) and -9 (Val352, Trp354, Lys292), Leu (P_2_) of Ac-DNLD-CHO is difficult to be accepted (Fig. 8). In contrast, since the Arg at the S_3-3 _subsites is conserved in all caspase family proteins (Fig. 4A), the interactions of Glu (P_3_) of Ac-DEVD-CHO with the S_3-3 _subsites are considered to decrease the selectivity while they increase the binding affinities of Ac-DEVD-CHO (Fig. 8, 2nd. column). The hydrophobic contacts between Val (P_2_) of Ac-DEVD-CHO and the S_2 _subsites are probably weaker than those between Leu (P_2_) of Ac-DNLD-CHO and the S_2 _subsites (Fig. 8). This prediction is supported by comparisons of the changes in Accessible Surface Area (ΔASA) between Leu (P_2_) and Val (P_2_), which are calculated to be 145 and 113 Å ^2^, respectively (Table 3). Because it is known that there is good correlation between the amount of nonpolar surface of higher ΔASA and hydrophobic interactions [36], the ΔASA of Leu (P_2_) of Ac-DNLD-CHO may bear to the potent binding affinity through the hydrophobic effect.

### Alanine replacement analysis for specific recognition of DNLD by caspase-3

The above computational docking experiments suggest that the specific recognition of DNLD sequence by caspase-3 is attributable to the notable hydrogen bond between Asn (P_3_) and Ser209 (S_3–4 _subsite).

To prove the significance of Ser209 in the interaction of the substrate Asn, the caspase-3 residue Ser209 was replaced with alanine by site-directed mutagenesis and the effect on recognition of DNLD was determined using MCA-fused peptide substrates. The substituted (S209A) and wild-type caspase-3 (active forms) were generated by coexpression of HA-p17 and HA-p12 subunits in *in vitro *translation system (Fig. 7E). The mutant caspase-3 cleaved Ac-DEVD-MCA as efficient as the wild-type. In contrast, the substitution (S209A) resulted in greater than 60% loss of cleavage activity on Ac-DNLD-MCA (compare Fig. 7C and 7D). These results strongly support the computational prediction that the specific interaction of DNLD with caspase-3 is attributable to the hydrogen bond between Asn (P_3_) and Ser209 in the S_3–4 _subsite.

### Specific interaction of Ac-DNLD-CHO with caspase-3

To examine the specificity of Ac-DNLD-CHO for caspase-3, the binding mode was further compared with Ac-DQTD-CHO and Ac-DMQD-CHO. Ac-DQTD-CHO, which inhibits caspases-3, -7, -8, and -9 activities with K_i_^app ^values of 1.27 nM, 21.8 nM, 9.75 nM, and 14.5 nM, respectively (Table 1), has a similar selectivity for these caspases as Ac-DEVD-CHO, and its potency is one-order of magnitude lower than that of Ac-DEVD-CHO (Fig. 1). The reasons for the poor selectivity of Ac-DQTD-CHO as compared with Ac-DNLD-CHO may be due to the formation of hydrogen bonds between Gln (P_3_) with the Arg207 (caspase-3), Asn88 (caspase-7), Arg258 (caspase-8), or Arg355 (caspase-9) as in the case of Ac-DEVD-CHO, and weak hydrophobic contacts with the S_2 _subsites of all caspases (Fig. 8, 3rd. column).

On the other hand, Ac-DMQD-CHO, which inhibits the activities of caspases-3, -7, -8, and -9 with K_i_^app ^values of 13.3 nM, >200 nM, >200 nM, and >200 nM, respectively (Table 1), has unique selectivity for caspase-3, but with low potency for all. This may be attributed to a lack of interaction between Gln (P_2_) and the S_2 _subsite (Fig. 8, 4th. column). Meanwhile, no side-chain hydrogen bonds between Met (P_3_) of Ac-DMQD-CHO and the S_3-3 _subsites of caspases-8, and -9 are formed. In the cases of caspases-3 and -7, the formation of hydrogen bonds between Met (P_3_) and the Arg (Arg207 for caspase-3 and Arg233 for caspase-7) in the S_3-3 _subsites is possible (Fig. 8, 4th. column). These results strongly support the specificity of Ac-DNLD-CHO for caspase-3. The biding modes used in this analysis are available in additional data file [see Additional file 1].

## Discussion

Ac-DNLD-CHO is the first reported example of a novel potent peptide inhibitor of caspase-3 [32]. The sequence was rationally designed by the structure-based computational APF method, and is so far not known in any natural caspase substrate. Furthermore, Ac-DNLD-CHO has been shown to inhibit caspase-3 as potently as Ac-DEVD-CHO, a well-known caspase-3 inhibitor. These observations suggest that the APF method is useful to design effective peptides with high binding affinities for target proteins. In the present study, we examined the specificity of Ac-DNLD-CHO for caspase-3 by inhibition kinetics analysis, computational docking studies and site-directed mutagenesis. In our *in vitro *assay system, Ac-DNLD-CHO inhibits caspase-3 (K_i_^app ^= 0.68 nM) more selectively than caspases-7, -8, and -9 (K_i_^app ^= 55.7, >200, and >200 nM, respectively). These observed K_i_^app ^values correlate well with the calculated free energies of binding (ΔG_calc_) by AutoDock between Ac-DNLD-CHO and caspases-3, -7, -8, and -9 (ΔG_calc _= -12.99, -11.99, -10.78, and -9.98 kcal/mol, respectively). In plots of the calculated ΔG_calc _values and experimentally observed K_i_^app ^values, a correlation coefficient of R = 0.75 was obtained. This implies that the AutoDock produces reliable binding conformations of tetrapeptides on caspase molecules and that it is suitable for the analysis of the binding modes of tetrapeptide/caspase complexes.

The docking studies data reveal the notable characteristics of Ac-DNLD-CHO binding to caspase-3 to be the hydrogen bonds between Asn (P_3 _position) and Ser209 in the S_3–4 _subsite of caspase-3, and the tightly hydrophobic contacts between Leu in the P_2 _position and the S_2 _subsite composed of three aromatic amino acids, Tyr204, Trp206, and Phe256. In the S_3 _subsite of caspase-3, Ser209 (S_3–4_) is highly preferred for the interaction with Asn (P_3_), although other residues such as Arg can be accommodated, as observed in the case of caspase-8 (Fig. 8, 1st. column). A weak side chain-side chain hydrogen bond is formed between Arg258 (S_3-1_) of caspase-8 and Asn (P_3_) rather than that formed between Ser209 (S_3–4_) of caspase-3 and Asn (P_3_). Hence, Asn in Ac-DNLD-CHO is the only peptide residue involved in a side-chain-specific hydrogen bond with Ser209 (S_3–4_) in the caspase-3 molecule.

The alignment of active site residues also supports these characteristic, and provides the information for speculating the binding ability of Ac-DNLD-CHO to other apoptotic caspases. The S_3–4 _subsites of caspases show considerable diversity in their amino acids and in their interaction with Asn (P_3_) of Ac-DNLD-CHO, which is only possible for caspase-3. In caspase-8, Asn (P_3_) is able to interact with Arg258 (S_3-1_) only when the conformation of the main-chain backborn is considerably changed (Fig. 8, 1st. column). Arg207, Arg233, Arg 413, and Arg355 (S_3-3 _subsites) in caspases-3, -7, -8, and -9, respectively, do not interact with Asn (P_3_). Meanwhile, the Glu in the P_3 _position of Ac-DEVD-CHO binds to Arg207 (S_3-3 _subsite) in caspase-3. The S_3-3 _subsites of caspases-3, -7, -8, and -9, however, are also conserved as Arg residues. Therefore, Glu (P_3_) probably interacts tightly with Arg (S_3-3 _subsites) in all of the caspases. Obviously, substitution of Glu with Asn in the P_3 _position results in a substantial increase in the selectivity of DNLD for caspase-3.

On the other hand, amino acid residues in the P_2 _positions of peptides such as Leu and Val are highly preferred in the hydrophobic pockets in S_2 _subsites except for caspases-8 and -9, as illustrated in Fig. 8. Furthermore, the S_2 _subsites of caspases-3 and -7 have three hydrophobic aromatic residues. The structural analyse combined with the inhibitory activities suggest a critical role for the hydrophobic interactions between Leu (P_2_) in Ac-DNLD-CHO and the S_2 _subsite of caspase-3, since the ΔASA of Leu (P_2_) is wider than Val (P2) of Ac-DEVD-CHO (Table 3). Furthermore, although the docking information obtained by the comparison of DNLD with DEVD is somewhat difficult to understand completely, the stereochemical configuration of the peptide is important for both binding and inhibitory activity, while the loss of hydrogen bonds and hydrophobic interactions are critical.

The specific inhibitory activity of Ac-DNLD-CHO against caspase-3 is also confirmed by the comparison of both inhibitory activities and binding modes with commercially available caspase-3 peptide inhibitors, Ac-DQTD-CHO and Ac-DMQD-CHO (Fig. 8). The Gln (P_3 _position) side chain of Ac-DQTD-CHO can make contact with Args in the S_3-3 _subsites of caspases-3, -7, -8, and -9. The Thr in the P_2 _position interacts with hydrophobic pockets in the S_2 _subsites in all caspases. Hence, Ac-DQTD-CHO shows less selectivity and a lower inhibitory activity than Ac-DNLD-CHO. On the other hand, for Ac-DMQD-CHO, the Met substitution in the P_3 _position, which might reduce its ability to form hydrogen bonds with Arg in S_3-3 _subsites of caspases-3 and -7, resulting in a substantial decrease in inhibitory activity (Table 1). The substitution Leu or Val by Gln in the P_2 _position results in a complete lack of interaction, thereby drastically decreasing the inhibitory activity. On the basis of these docking studies, the specific inhibitory activity of Ac-DNLD-CHO on caspase-3 is strongly suggested to be due to the selective hydrogen bond between Asn (P_3_) and Ser209 in the S_3–4 _subsite, and the tightly hydrophobic contacts of Leu (P_2_) with the aromatic amino acids Tyr204, Trp206, and Phe256 in the S_2 _subsite.

Furthermore, we performed critical examinations of the sequence specificity of DNLD using fluorogenic MCA-peptide substrates, since in general, recognition of peptide sequence is detected more stringently in substrates than inhibitors. As expected, the fluorogenic substrate Ac-DNLD-MCA exhibited high selectivity for caspase-3 over caspases-7, -8, and -9. This implies that Ac-DNLD-MCA is useful for the precise measurement of the activity of caspase-3 in crude apoptotic cell extracts. In site-directed mutagenesis studies, the substituted (S209A) caspase-3 considerably decreased the ability to cleave Ac-DNLD-MCA (Fig. 7C). This is consistent with the computationally predicted binding mode of Ac-DNLD-CHO on caspase-3 (Fig. 7A). Obviously, the hydrogen bond between the caspase-3 residue Ser209 and the substrate Asn (N) plays a critical role in the specificity of the peptide.

Recently, to monitor the time course of caspase-3 activation during apoptosis at the cellular level, an expressed fusion protein containing cyan fluorescent protein (CFP) linked by a short peptide possessing the caspase-3 cleavage sequence, DEVD, to yellow fluorescent protein (YFP) (i.e. CFP-DEVD-YFP) has been utilized [37]. The protein undergoes fluorescence resonance energy transfer (FRET) between CFP and YFP. When caspase-3 is activated, the DEVD sequence is cleaved and FRET is reduced [37]. By using the DNLD sequence, a more precise measurement is possible.

Previous studies have indicated that peptide-based caspase inhibitors are effective in animal models of ALS [21], sepsis [22], and hypoxic-ischemic brain injury [23]. Some inhibitors, however, have poor whole cell activity due to limited cell permeability [38]. In this study, although the *in vivo *use of Ac-DNLD-CHO was not performed, the activity would be enhanced by modifying the DNLD sequence with benzylcarboxy, methyl ester, and fluoromethyl ketone (FMK) (i.e. Z-D(OMe)NLD(OMe)-FMK) or protein transduction domains (PTDs), such as the TAT protein [39], Antennapedia homeodomain [40], or arginine-rich peptides [41] (i.e. PTD+Linker+DNLD-CHO). Furthermore, many peptide-mimetic caspase-3 inhibitors have been prepared based on the DEVD tetrapeptide. [38,42,43]. As has been suggested previously [32], the optimization of a binding peptide sequence for a target protein, such as DNLD, includes obtaining sufficient information about its subsequent conversion to small molecular non-peptidic compounds. The DNLD tetrapeptide characterized here, with a molecular mass of about 500, is within reach of small molecule design and can serve as a minimal model for a caspase specific inhibitor. Therefore, the optimized peptide-based design of small molecule mimetics would be expected to be possible by using the configuration with the lowest ΔG. In developing non-peptidic caspase-3-specific inhibitors, the DNLD tetrapeptide provides a valuable starting point for the design of specific peptide-mimetics and their optimization. For example, a unique side chain containing an amide is present at the P_3 _position of DNLD, suggesting that this is a critical target region in the design of caspase-3-specific small molecules and potential therapeutic agents.

## Conclusion

On the basis of our results, we conclude that Ac-DNLD-CHO is a reliable, potent and selective inhibitor of caspase-3. The binding site for DNLD comprises the same active pockets of caspase-3 that are known to kind other inhibitory peptides such as DEVD, DQTD, and DMQD. The substituted residues Asn and Leu in the P_3 _and P_2 _positions, respectively, play important roles in both the selectivity and potency for caspase-3. The specific inhibitory effect on caspase-3 suggests that this inhibitor could become an important tool for investigations of the biological function of caspase-3. Ac-DNLD-MCA could also be useful in determining whether caspase-3 acts in cells that respond to various apoptotic stimuli such as drugs and viruses. Furthermore, Ac-DNLD-CHO may be an attractive lead compound to generate novel effective non-peptidic pharmaceuticals for caspase-mediated apoptosis diseases, such as neurodegenerative disorders and viral infection diseases.

## Methods

### Materials

Ac-DNLD-CHO and Ac-DNLD-MCA were synthesized by Peptide Institute, Inc (Osaka, Japan). Ac-DEVD-CHO, Ac-DQTD-CHO, Ac-DMQD-CHO, Ac-DEVD-MCA, Ac-IETD-MCA, and Ac-LEHD-MCA were purchased from Peptide Institute, Inc. Recombinant human caspases-3, -7, -8, and -9 were from Calbiochem.

### Caspase assay and inhibition

The activities of caspases-3 and -7 were measured using Ac-DEVD-MCA as the substrate. The activities of caspases-8 and -9 were measured using Ac-IETD-MCA and Ac-LEHD-MCA, respectively. To compare the inhibitory potencies of Ac-DNLD-CHO, Ac-DEVD-CHO, Ac-DQTD-CHO, and Ac-DMQD-CHO against caspases-3, -7, -8, and -9, one unit (the amount of enzyme that cleaves 1 nmol of the substrate sequence per hr) of each active recombinant human caspase was incubated with various concentrations of a peptide inhibitor in assay buffer (100 mM NaCl, 50 mM HEPES, 10 mM DTT, 1 mM EDTA, 10% glycerol, and 0.1% CHAPS, pH 7.4) at 37°C for 30 min with shaking in a 96-well assay plate (CORNING). Then, caspase substrate was added to each well to a final concentration of 200 μM and the liberation of MCA was monitored continuously at 37°C using a 96-well plate reader Wallace 1420 ARVOsx (PerkinElmer) with an excitation wavelength of 390 nm and an emission wavelength of 460 nm.

### Ac-DNLD-MCA and Ac-DEVD-MCA cleavage assay

To characterize the potency and selectivity of Ac-DNLD-MCA for caspases-3, -7, -8, and -9, in vitro caspase activity assays were performed. One unit (the amount of enzyme that cleaves 1 nmol of substrate per hr) of each active recombinant human caspase was added to a reaction mixture containing 200 μM Ac-DNLD-MCA or Ac-DEVD-MCA in assay buffer (100 mM NaCl, 50 mM HEPES, 10 mM DTT, 1 mM EDTA, 10% glycerol, and 0.1% CHAPS, pH 7.4). Then, the liberation of MCA was monitored continuously at 37°C using a 96-well plate reader Wallace 1420 ARVOsx (PerkinElmer) with an excitation wavelength of 390 nm and an emission wavelength of 460 nm.

### Construction of expression vectors of HA tagged p17 or p12 subunits of caspase-3

Human cDNA fragments containing p17 or p12 subunits of caspase-3 were generated by PCR from Jurkat cells. The primers used were p17, 5'-TCTGGAATATCCCTGGACAA C-3' (sense) and 5'-TTAGTCTGTCTCAATGCCACAGTC-3' (antisense); p12,5'-AGTGGT GTTGATGATGACATG-3' (sense) and 5'-TTAGTGATAAAAATAGAGTTC-3' (antisense). The amino-terminus of p17 or p12 were tagged with the HA epitope by PCR using the primers, 5'-CTCGAGCCACCATGTACCCATAC-3' (sense) and 5'-AGCGTAGTCTGGGAC GTCGTATGGGTACAT (antisense). The amino-terminus of HA tag includes XhoI site, and the site flanking the coding sequence is shown in bold letter. The PCR products were then subcloned into pcDNA3 B (Invitrogen) at the EcoRV site. After confirming the sequences, the inserts were excised by XhoI digestion and recloned into the XhoI site of pURE4 (Post Genome Institute Co., Ltd.).

### Site-directed mutagenesis of p12 subunit

Site-directed mutagenesis of p12 subunit (S209A) was introduced using PCR. The following primers were used for the preparation of the mutant, 5'-AGTGGTGTTGATGA TGACATG-3' (position 176–208, sense) and 5'-ATTTCGCCAAGAATAATAACC-3' (position 176–208, antisense); 5'-GCAAAGGATGGCTCCTGGTTCATC-3' (position 209–277, sense) and 5'-TTAGTGATAAAAATAGAGTTC-3' (position 209–277, antisense). The mutated site (Ser209→Ala) was shown in bold letter. After each fragments were amplified and ligated, they were tagged with HA and subcloned into pcDNA3 B (Invitrogen) at the EcoRV site. After comfirming the sequences, the inserts were excited by XhoI digestion and recloned into the XhoI site of pURE4 (Post Genome Institute Co., Ltd.)

### Generation of active caspase-3

Active caspase-3 was generated by coexpression of HA-p17 and HA-p12 (wild type or mutant) subunits *in vitro *using PURESYSTEM classic II (Post Genome Institute Co., Ltd.) according to the manufacturer's instructions.

### Western blot analysis

Two μl aliquots of proteins (wild type or mutant) were subjected to 15% SDS/polyacrylamide gel and transferred onto PVDF transfer-membranes. Blots were blocked in TBST [20 mM Tris-HCl (pH 8.0), 400 mM NaCl, and 0.05% (w/v) Triton X-100] containing 2.5% (w/v) BSA for 1.5 h and probed with anti-HA antibody (Bethyl Laboratories, Inc.). After the membrane were washed with TBST, retained antibody was detected with anti-rabbit IgG-alkaline phosphatase conjugate and a Prote Blot Western detection kit (Promega).

### Construction of phylogenetic tree

The amino acid sequences of the seven full-length human caspases retrieved from the Swiss-Prot database (accession numbers: caspase-2, P42575; caspase-3, P42574; caspase-6, P55212; caspase-7, P55210; caspase-8, Q14790; caspase-9, P55211; caspase-10, Q92851) were aligned using the Clustal W multiple alignment program [44], and then adjusted manually. Multiple alignment using the BLOSUM matrix series [45], a gap opening penalty of 11.0, and a gap extension penalty of 0.05 were chosen for aligning the sequences. Based on the alignment, a phylogenetic tree was constructed by the neighbor-joining (NJ) algorithms [35]. To establish a classification for the active sites of caspases, phylogenetic analysis of the active site residues was conducted by the NJ algorithm (exclude positions with gaps). Bootstrap analysis was performed on 1000 random samples and analyzed by the Clustal W [44]. The NJ analyses were displayed using NJ plot [46].

### Computational molecular modelling

Molecular visualization was carried out in a DS ViewerPro (Accelrys, Inc., San Diego, CA). The coordinates of caspases-3, -7, -8, and -9 were obtained from the Protein Data Bank (PDB) (codes 1PAU, 1F1J, 1F9E, and 1JXQ). Water was removed from the PDB files. The crystal structures of caspases-3, -7, -8, and -9 include coordinates of Ac-DEVD-CHO, Ac-DEVD-CHO, Z-DEVD-CHO, and Glu-Val-Dehydroxymethylaspartic acid, respectively, as inhibitors. Since it is known that Ac-DEVD-CHO potently inhibits the activities of caspases [31], we constructed the models of the Ac-DEVD-CHO/caspase complex. To construct the complex structure of caspase-8/Ac-DEVD-CHO, the benzyloxy-carbonyl group of Z-DEVD-CHO was displaced by the acetyl group on the DS ViewerPro. The complex structure of Ac-DEVD-CHO/caspase-9 was constructed by superposition with that of the Ac-DEVD-CHO/caspase-8. The superposition was performed using the McLaghlan algorithm [47] as implemented in the ProFit program [48]. The geometries of these complex structures were subsequently optimized using the GROMACS program [49,50]. The coordinate of Ac-DEVD-CHO was converted to a GROMACS topology file by the Dundee PRODRG2 Server [51]. Energy minimizations of the complex structures using a GROMACS forcefield were performed with the steepest descent algorithm.

### Computational molecular docking

Molecular docking was carried out using AutoDock3.0 [34] on a COMPAQ Alphastation DS20E (double 833 MHz processors and 1024 MB of memory). The binding free energy scoring function in the AutoDock is based on an empirical function derived by linear regression analysis of a large set of diverse protein-ligand complexes with known inhibition constants. There are many successful examples of structures of protein-ligand systems studied by the AutoDock program [52,53]. The docking energy grid (grid maps with 60 × 60 × 60 points, grid spacing 0.375 Å) was produced with the AutoGrid program [34]. The inhibitor centers in the complex structures were positioned at the grid center. The Lamarckian Genetic Algorithm was utilized, and energy evaluations were set at 3 × 10^6^. Each simulation was performed a total of 20 times. Other parameters were default values. The initial conformations of caspase-3 inhibitors, Ac-DNLD-CHO, Ac-DEVD-CHO, Ac-DQTD-CHO, and Ac-DMQD-CHO, were built using coordinates that replaced the side chains of the Ac-DEVD-CHO in the complex structures with the side chains of the inhibitors. Rotational bonds in the inhibitors were assigned with the program AutoTors [34]. All torsions except the peptide bonds were unconstrained during the docking. Based on the docking data, the lowest-energy docking mode was used to analyze the potency and selectivity of the caspase-3 peptide inhibitors.

## Authors' contributions

AY participated in study design, and wrote the manuscript. AY and ST dealt with the computational aspects in the implementation. JS, NO and RT assisted with study design and performed caspase inhibition assays. SS, NO and TK performed the construction of expression vectors and site-directed mutagenesis. SIT conceived the study and participated in its design and coordination. All authors read and approved the final manuscript.

## Supplementary Material

Additional File 1 ZIP file is a pdb format that is compressed. It contains caspase-3/peptide complexes (casp3_pep.pdb), caspase-7/peptide complexes (casp7_pep.pdb), caspase-8/peptide complexes (casp8_pep.pdb), and caspase-9/peptide complexes (casp9_pep.pdb). Click here for file
